# Cardiac Hepatoma: An Unlikely Cause of Malignant Cardiac Metastatic Disease

**DOI:** 10.7759/cureus.69202

**Published:** 2024-09-11

**Authors:** Sonia Vicenty-Rivera, Victor H Molina-Lopez, Alex P Rodriguez

**Affiliations:** 1 Cardiology, Bruce W. Carter VA Healthcare System, Miami, USA; 2 Cardiology, VA Caribbean Healthcare System, San Juan, PRI

**Keywords:** non-alcoholic steatohepatitis, metabolic dysfunction-associated fatty liver disease, right heart failure, cardiac tumors, hepatoma

## Abstract

Cardiac hepatoma is an extremely rare presentation of tumor metastasis. We present a case of an 80-year-old male who presented with symptoms of heart failure and was subsequently diagnosed with cardiac metastatic tumors. This case report highlights the diagnostic challenges and management options associated with this rare entity.

## Introduction

Cardiac tumors are rare and are classified as primary or secondary (metastatic). Secondary cardiac tumors are seen 20-30 times more frequently than primary ones [1]. Cardiac metastasis can occur in up to 10% of all cancer patients. The most common cancers that metastasize to the heart are lung, breast, kidney, leukemia, and melanoma. Cardiac metastasis can occur on the right side in 20-30% of cases, on the left side in 10-33% of cases, bilaterally or diffusely in 30-35% of cases, and on the endocardium or inside the heart cavity in 5% of cases [1,2]. Most cardiac tumors present a combination of signs and symptoms such as arrhythmias, embolic events, and heart failure. The severity of symptoms will depend on the location and size of the tumor, regardless of its histological type [3,4]. Heart metastasis is rare, occurring in 5-10% of patients with advanced hepatocellular carcinoma (HCC). Its presence indicates advanced disease and a poor prognosis [5-7]. Patients with advanced HCC often exhibit symptoms of heart failure due to flow obstruction or thromboembolism, in addition to symptoms related to chronic liver disease [8].

## Case presentation

An 80-year-old male with a history of persistent atrial fibrillation on Apixaban, arterial hypertension, multiple dysplastic nevi, type 2 diabetes mellitus, hypothyroidism on hormone replacement therapy, chronic mild thrombocytopenia, intermittent liver enzyme elevation, and hypothyroidism on levothyroxine replacement presented to the emergency department (ED) with progressively worsening exertional dyspnea.

The patient reported social alcohol use, consuming three to four beers twice a week. He had no tattoos, no history of blood transfusions, and no prior diagnosis of liver disease. The patient was previously active with no physical limitations until six months before admission, when he experienced an unintentional 30-pound weight loss, progressing to dizziness upon standing, exertional fatigue, and severe shortness of breath with minimal activity. Additionally, he noted lower extremity swelling but denied fevers, abdominal pain, chest discomfort, hematuria, or rectal bleeding. Given the limiting symptoms, the patient sought medical attention at the ED.

**Figure 1:**
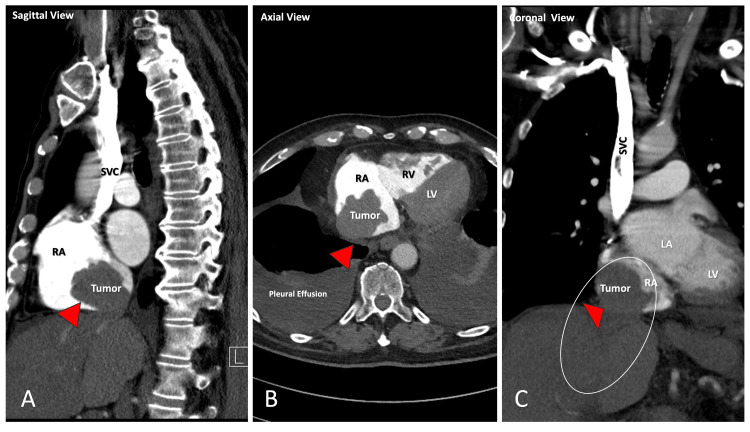
Initial chest CT angiography (CTA) imaging demonstrating a large lobulated filling defect in the RA suspicious for malignancy or tumor thrombus in (A) sagittal, (B) axial, and (C) coronal views IVC: inferior vena cava; SVC: superior vena cava; RA: right atrium; RV: right ventricle; LA: left atrium; LV: left ventricle

Bedside physical examination revealed no evidence of jugular venous distention. Cardiac auscultation noted an irregular heart rhythm without audible murmurs, gallops, or rubs. Pulmonary auscultation was clear, with no abnormal findings. The patient exhibited 2+ pitting edema in the lower extremities. Laboratories on admission were remarkable for elevated liver enzymes (over three times the upper limit of normal), bilirubin, and alkaline phosphatase (Table 1). Twelve-lead electrocardiogram showed atrial fibrillation, low voltage QRS, poor precordial R wave progression, and nonspecific T-wave abnormality now evident in inferior leads. A portable chest X-ray showed bilateral lower lobe consolidation without pulmonary edema. Nevertheless, there was a small bilateral pleural effusion and mild cardiomegaly. A chest computerized tomographic angiography (CTA) was performed to rule out pulmonary embolism; it revealed no emboli but identified a significant, lobulated filling defect in the right atrium, appearing to emanate from the inferior vena cava (IVC), measuring approximately 5.1 x 4.5 cm in transverse dimensions and 4.5 cm in the axial dimension (Figure 1, A-C). This finding was new and was not previously seen on a chest CT performed six months before the symptom presentation. Additionally, the limited evaluation indicated cirrhotic changes in the liver parenchyma.

**Table 1:** Liver panel and enzymes during hospitalization AST: aspartate aminotransferase; ALT: alanine aminotransferase

Hepatic Labs	D0	D1	D4	D6	D8	D10	D25
AST (U/L)	138	110	124	155	142	189	161
ALT (U/L)	67	52	59	73	67	91	78
Alk. Phos (U/L)	217	183	203	211	214	265	260
Total bilirubin (mg/dl)	2.0	1.8	1.6	1.4	1.6	1.8	14.1
Albumin (g/dl)	3.3	2.9	2.8	2.7	2.6	2.6	2.7
Total protein (g/dl)	7.2	6.8	6.3	6.1	5.9	6.2	6.5

**Figure 2:**
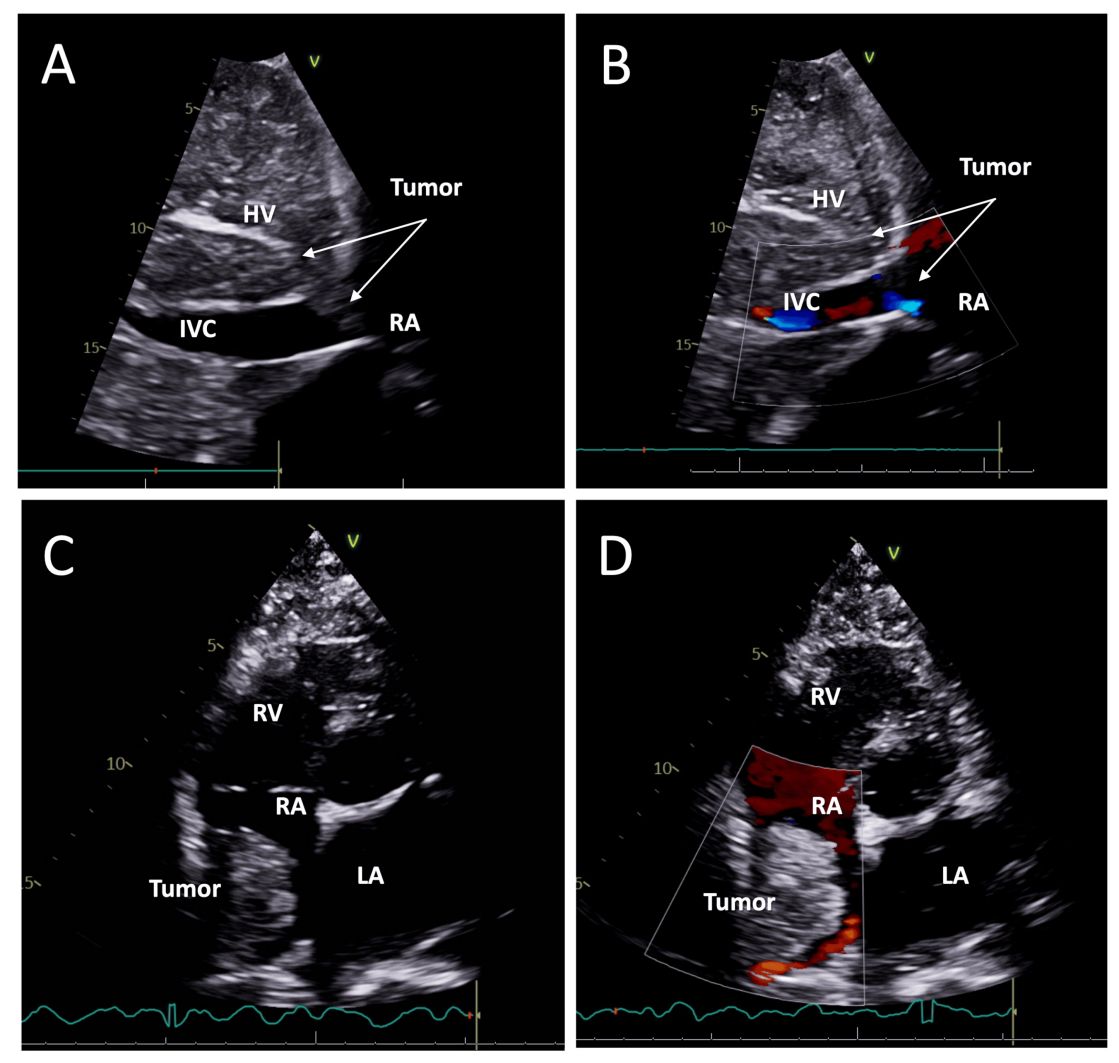
TTE revealed a large RA mass extending from the HV and IVC into the RA Subxiphoid view demonstrating a mass projecting from the HV into the IVC and RA on B-mode (A) and with color flow Doppler (B). Apical 4-chamber view showing a large RA mass on B-mode (C) and with color flow Doppler (D). TTE: transthoracic echocardiogram; IVC: inferior vena cava; RA: right atrium; RV: right ventricle; LA: left ventricle; HV: hepatic vein

A transthoracic echocardiogram (TTE) revealed a large right atrial mass extending from the hepatic veins into the right atrium (Figure 2, A-D). There is a low normal left ventricle systolic function with an ejection fraction of 50-55%. Right atrial mass measuring 3.5 x 3.5 cm in diameter with echo-contrast agent (Optison®) uptake is suggestive of vascularity perfusion. The mass appears close to the tricuspid valve without apparent invasion or hemodynamic stenosis and only mild regurgitation. The IVC was normal in diameter and demonstrated a <50% inspiratory collapse, suggesting an estimated right atrial pressure of 8 mmHg.

**Figure 3:**
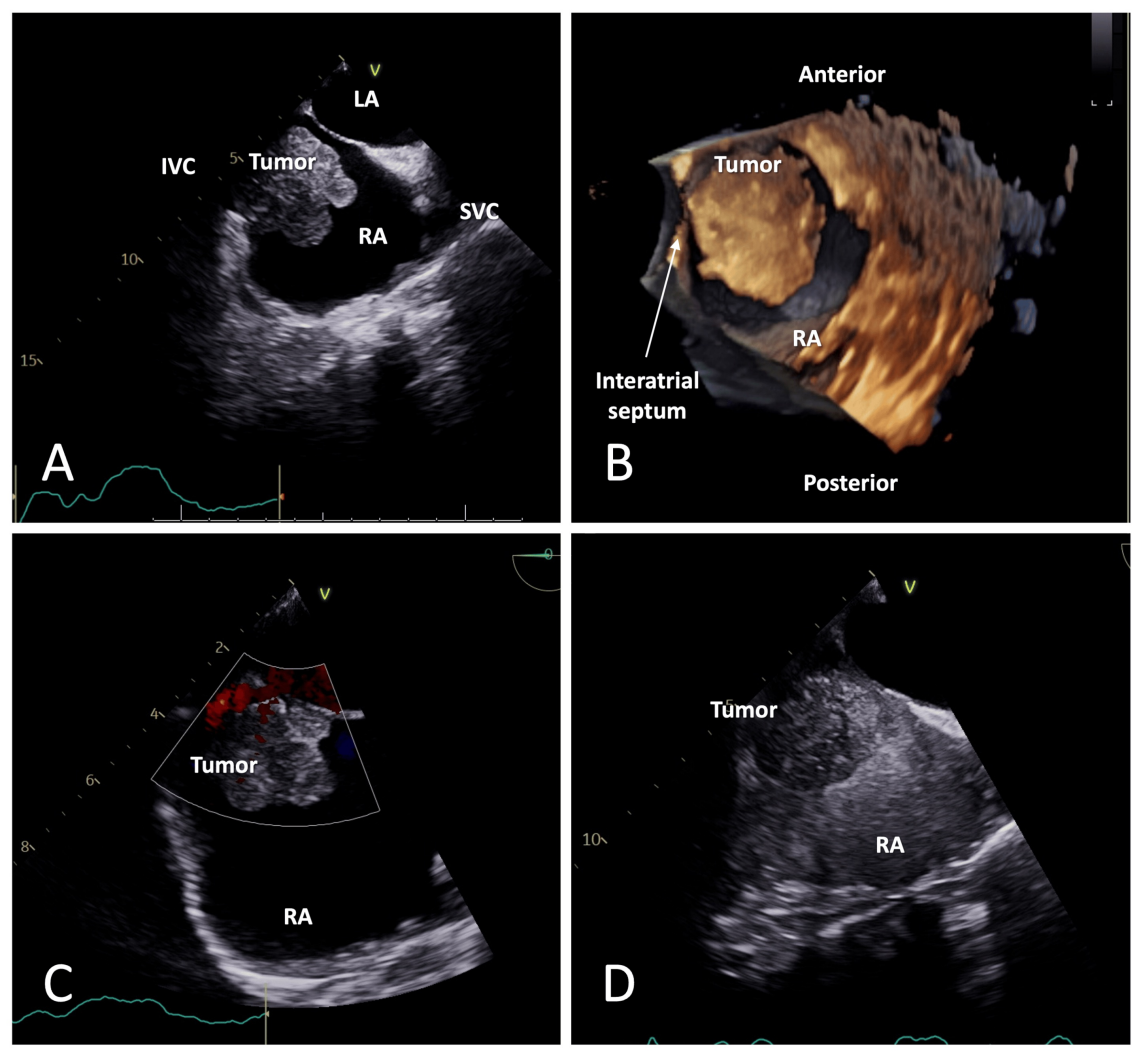
TEE evaluation of the RA mass (A) TEE evaluation demonstrating an RA mass projecting through the IVC in mid-esophageal bicaval view. (B) 3D imaging with post-processing demonstrating the RA mass in the RA *en face*. (C) Color flow interrogation without prominent vascularity. (D) Non-enhancing mass on contrast-enhanced echocardiography suggests a poorly vascularized mass. TEE: transesophageal echocardiogram; SVC: superior vena cava; IVC: inferior vena cava; RA: right atrium; LA: left atrium

Transesophageal echocardiography (TEE) was performed to better delineate the mass and its interaction with the tricuspid valve. It confirmed a large right atrial mass extended from the IVC and attached to the free wall of the right atrium (Figure 3, A-D). The mass was not leading to any hemodynamic obstruction through the tricuspid valve.

Laboratory evaluation revealed an elevated alpha-fetoprotein level of 21,373 ng/ml. The workup for hepatitis and human immunodeficiency virus (HIV) returned normal findings. Indicators of synthetic liver dysfunction included low platelets, elevated total bilirubin, and an increased internationalized normalized ratio (INR). The patient had risk factors for metabolic dysfunction-associated fatty liver disease (MASLD) with associated liver cirrhosis, reflected by a model for end-stage liver disease (MELD) score of 3.0.

**Figure 4:**
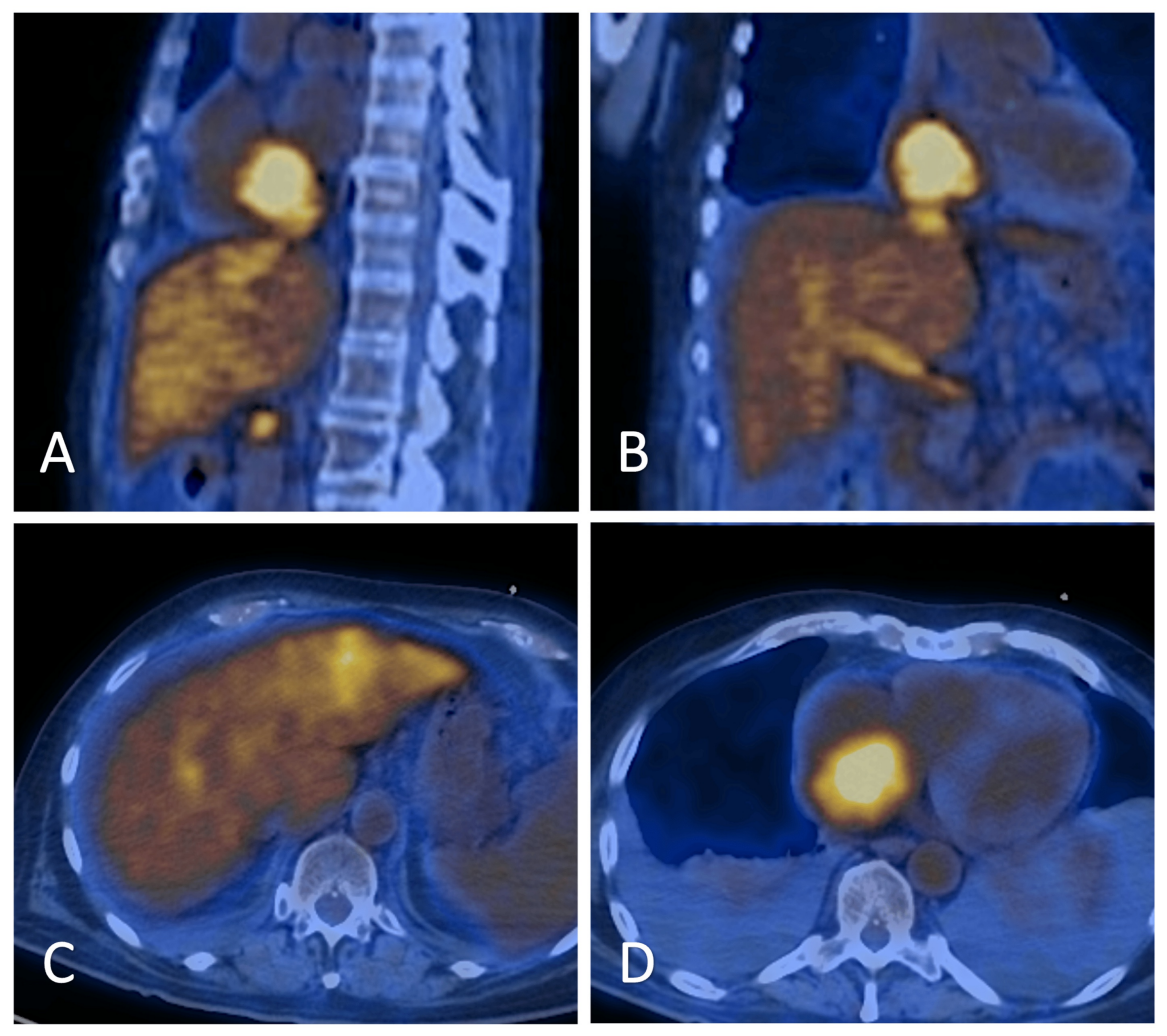
PET/CT demonstrating an hepatic mass with HV and RA extension The patient underwent imaging using PET/CT after receiving an intravenous injection of 12.9 mCi of F-18. The scan data, acquired from the brain to the upper thigh, was analyzed in attenuation-corrected axial (A), coronal (B), and transaxial (C & D) planes. The imaging revealed a large, lobulated mass within the RA (A, B, D) extending into the IVC, left HV, and middle HV. This mass demonstrated moderately intense metabolic activity, with a maximum standardized uptake value (SUV max) of 7.1, raising concerns for tumor thrombus. Additionally, the liver exhibited cirrhotic morphology with heterogeneous metabolic activity in the left hepatic lobe (C). PET: positron emission tomography; CT: computed tomography; IVC: inferior vena cava; HV: hepatic vein; RA: right atrium

A positron emission tomography-computed tomography (PET-CT) scan was performed to assess the extent of metastatic disease, revealing a large lobulated right atrial mass extending into the IVC, left hepatic vein, and possibly the middle hepatic vein, with a standardized uptake value (SUV) max of 7.1. These imaging findings indicated high metabolic activity and cirrhotic liver morphology, favoring a diagnosis of hepatocellular malignancy with tumor thrombus (Figure 4, A-C).

**Figure 5:**
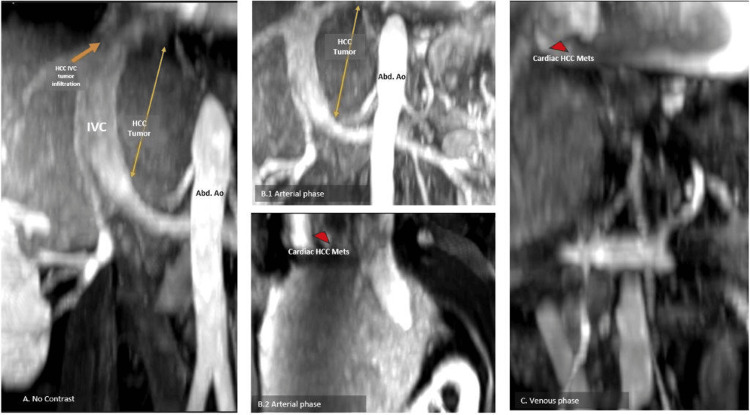
Triple-phase MRI of the liver with gadolinium enhancement demonstrating a hepatic mass with extensive tumor thrombus occluding essentially all the intrahepatic portal venous branches, supra-hepatic inferior vena cava, and right atrium, compatible with LR-TIV Liver MRI with intravenous contrast and a liver-specific protocol revealed an infiltrative liver mass occupying nearly the entire left lobe and parts of the central right lobe (A, B.1, and C). The mass extended as a tumor thrombus into the intrahepatic portal vein branches of both lobes, the main portal vein up to the portosplenic confluence, and further involved the left HV, extending to the suprahepatic IVC and the RA (B1 and 2). These imaging findings are consistent with HCC, classified as LI-RADS TIV. Additionally, a LI-RADS 4 lesion measuring 17 mm was identified in hepatic segment 5. The imaging also revealed sequelae of portal hypertension, including splenomegaly, a splenorenal shunt, a recanalized periumbilical vein, gastric varices, and small-volume ascites. MRI: magnetic resonance imaging; IVC: inferior vena cava; RA: right atrium; HV: hepatic vein; HCC: hepatocellular carcinoma; LI-RADS TIV: liver imaging reporting and data system tumor in vein

A multisequence, multiplanar abdominal magnetic resonance imaging (MRI) with contrast enhancement using a liver protocol was performed (Figure 5). In Figure 5, liver MRI shows a hyperintense T2-weighted signal of a large infiltrative mass measuring 12 cm in its maximum linear diameter, involving nearly the entire left hepatic lobe with heterogeneous arterial phase hyperenhancement and delayed phase washout and capsular enhancement, all compatible with hepatocellular carcinoma. The tumor thrombus extended to all intrahepatic portal venous branches of both the right and left hepatic lobes as well as the main portal vein back to the portal splenic confluence. There was also tumor thrombus extension occluding the left hepatic vein and non-occlusive thrombus extending cephalad to the intrahepatic and suprahepatic IVC up to the right atrium. Overall, the extension of the infiltrative mass was compatible with HCC with the macrovascular invasion of the portal and/or hepatic vein (LR-TIV). 

Hematology/oncology thoroughly evaluated the patient's condition. Their recommendation was to start on palliative chemotherapy possibly with Atezolizumaba and Bevacizumab with referral to palliative and hospice care. Nevertheless, due to his advanced age and terminal disease, the patient decided against chemotherapy treatment and continued with palliative/hospice care until his demise.

## Discussion

Hepatocellular carcinoma (HCC) is the most common type of primary liver cancer and is the fourth leading cause of cancer-related death. It is considered the second most lethal cancer after pancreatic carcinoma, with a five-year survival rate of 18% [3]. According to the North American Association of Central Cancer Registries, 41,210 new cases of HCC were estimated to have been diagnosed in the US in 2023. The incidence rate of HCC has tripled over the past four decades, but since 2015, the increase has stabilized. It is the most frequent cause of death in patients with cirrhosis due to chronic liver injury with fibrosis and inflammation, with an annual incidence between 2-4% [7]. The clinical presentation of HCC depends on the tumor stage and the background liver tissue cirrhosis.

Symptoms related to HCC present in advanced stages, and most patients already have symptoms related to their chronic liver disease, which will be worsening at the time of cancer diagnosis [8]. Most of the symptoms described by the patient are nonspecific and can range from general weakness, tiredness, nausea, abdominal pain, weight loss, unintentional weight loss, and vomiting. The most prominent features can be jaundice, increased abdominal girth with ascites, and easy bruising or bleeding secondary to coagulopathies. On the other hand, some patients with HCC can present paraneoplastic symptoms of hypoglycemia, erythrocytosis, hypercalcemia, diarrhea, and cutaneous findings, such as pemphigus foliaceous, pityriasis rotunda, dermatomyositis, and the aggressive onset of multiple seborrheic keratoses with an inflammatory base, also known as Leser-Trelat sign. Our case is quite interesting instead of its silent presentation, albeit radiological evidence of cirrhotic liver disease due to non-alcoholic steatohepatitis (NASH). Furthermore, the patient's symptoms were mostly dyspnea and volume overload without any signs or symptoms of chronic liver disease. The most likely reason for his dyspnea and volume overload was due to intermittent tricuspid valve/right ventricular inflow obstruction [9].

In our case, the diagnosis of hepatocellular carcinoma (HCC) was made based on imaging features and a high level of alpha-fetoprotein. HCCs are highly vascular tumors commonly found in patients with fibrotic or cirrhotic livers [10,11]. The increased vascularity is the main histological characteristic that enhances the accuracy of multiphasic computed tomography (CT) and magnetic resonance imaging (MRI) as primary diagnostic tests [12]. Both imaging studies are evaluated using the Liver Imaging Reporting and Data System (LI-RADS) algorithm for HCC diagnosis [13]. Therefore, liver biopsy is no longer necessary for a definitive diagnosis when precise imaging results are obtained. It is only required in cases where imaging CT/MRI results are inconclusive and a definite diagnosis is needed, particularly when patients do not show evidence of cirrhosis [14].

Overall, HCC survival is poor, with a five-year relative survival rate of 18.4% and only 2% for metastatic hepatocellular carcinoma [15]. HCC usually metastasizes, which has been reported in up to 15-17% of patients with mostly lung, abdominal lymph node, bone, and adrenal gland involvement in that order [15]. Autopsy studies are rare and can occur with HCC cardiac metastasis occurring at 2.7-4.1% incidence of atrial metastases [16,17]. HCC cardiac metastasis occurs through direct tumor extension to the heart through the hepatic vein and inferior vena cava. There are no randomized controlled studies nor clear guidelines for advanced HCC metastasis involving the heart but a handful of case reports. Therefore, the management of advanced HCC includes a combination of surgical tumor resection and thrombus extraction, +/- liver transplantation, and systemic chemotherapy [13]. Nevertheless, despite treatment, median survival remains poor in patients with distant metastasis (eight months), and it is much worse in cardiac metastasis with one to four-month survival despite treatment. Palliative care becomes the cornerstone for symptom relief in advanced HCC patients with the main aim to control pain, depression/anxiety, fatigue, nausea, weight loss, nausea, ascites, shortness of breath, and skin problems [18]. JY Chang et al. published a small case series in which patients had advanced HCC with distant metastasis to the IVC/RA; thalidomide was administered for four weeks as a palliative measure for symptom control [19]. In their group of patients, not only was there symptom improvement but also a decrease in the alfa-fetoprotein level and tumor burden, demonstrating an improvement in outcome (around 15 months survival). In recent years, the landscape of advanced HCC has changed, with multiple ongoing trials evaluating the immune checkpoint inhibitors in association with anti-angiogenic agents. As of 2020, atezolizumab and bevacizumab have been shown to improve survival compared to sorafenib, which was validated. This is considered to be the first-line treatment for advanced hepatocellular carcinoma [20].

## Conclusions

HCC cardiac metastasis is a rare presentation with limited clinical presentation until advanced disease. The diagnostic process relies mostly on imaging studies and is interpreted using the liver imaging reporting and data system (LI‐RADS) algorithm for HCC diagnosis. Liver biopsy is deferred for cases with unclear imaging findings. Treatment involves a combination of therapies, which depends on the availability of locoregional and local expertise. Palliative care is imperative for patient management.
